# Electronic Cigarettes Efficacy and Safety at 12 Months: Cohort Study

**DOI:** 10.1371/journal.pone.0129443

**Published:** 2015-06-10

**Authors:** Lamberto Manzoli, Maria Elena Flacco, Maria Fiore, Carlo La Vecchia, Carolina Marzuillo, Maria Rosaria Gualano, Giorgio Liguori, Giancarlo Cicolini, Lorenzo Capasso, Claudio D'Amario, Stefania Boccia, Roberta Siliquini, Walter Ricciardi, Paolo Villari

**Affiliations:** 1 Department of Medicine and Aging Sciences, University of Chieti, via dei Vestini 5, 66013, Chieti, Italy; 2 Local Health Unit of Pescara, Via Renato Paolini 47, 65100, Pescara, Italy; 3 "University G. d'Annunzio" Foundation, Via Colle dell'Ara, 66013, Chieti, Italy; 4 Regional Healthcare Agency of Abruzzo, Via Attilio Monti 9, 65127, Pescara, Italy; 5 Department "G. F. Ingrassia"- Hygiene and Public Health, University of Catania, Piazza Università 2, 95131, Catania, Italy; 6 Department of Clinical Sciences and Community Health, University of Milan, Via Vanzetti 5, 20133, Milan, Italy; 7 Department of Public Health and Infectious Diseases, Sapienza University of Rome, Viale Regina Elena 324, 00161, Roma, Italy; 8 Department of Public Health Sciences, University of Turin, Via Santena 5/bis, Turin, 10124, Italy; 9 Department of Movement Sciences and Wellbeing, University Parthenope of Neaples, Via Medina 40, 80133, Napoli, Italy; 10 Local Health Authority of Lanciano-Vasto-Chieti, Via Martiri Lancianesi, 66100, Chieti, Italy; 11 Institute of Public Health, Università Cattolica del Sacro Cuore, Rome, Largo Francesco Vito, 1, 00168, Roma, Italy; 12 Italian Institute of Health, Viale Regina Elena 299, 00161, Rome, Italy; Legacy, Schroeder Institute for Tobacco Research and Policy Studies, UNITED STATES

## Abstract

**Objective:**

To evaluate the safety and efficacy as a tool of smoking cessation of electronic cigarettes (e-cigarettes), directly comparing users of e-cigarettes only, smokers of tobacco cigarettes only, and smokers of both.

**Design:**

Prospective cohort study. Final results are expected in 2019, but given the urgency of data to support policies on electronic smoking, we report the results of the 12-month follow-up.

**Data Sources:**

Direct contact and structured questionnaires by phone or via internet.

**Methods:**

Adults (30–75 years) were included if they were smokers of ≥1 tobacco cigarette/day (tobacco smokers), users of any type of e-cigarettes, inhaling ≥50 puffs weekly (e-smokers), or smokers of both tobacco and e-cigarettes (dual smokers). Carbon monoxide levels were tested in a sample of those declaring tobacco smoking abstinence.

**Main Outcome Measures:**

Sustained smoking abstinence from tobacco smoking at 12 months, reduction in the number of tobacco cigarettes smoked daily.

**Data Synthesis:**

We used linear and logistic regression, with region as cluster unit.

**Results:**

Follow-up data were available for 236 e-smokers, 491 tobacco smokers, and 232 dual smokers (overall response rate 70.8%). All e-smokers were tobacco ex-smokers. At 12 months, 61.9% of the e-smokers were still abstinent from tobacco smoking; 20.6% of the tobacco smokers and 22.0% of the dual smokers achieved tobacco abstinence. Adjusting for potential confounders, tobacco smoking abstinence or cessation remained significantly more likely among e-smokers (adjusted OR 5.19; 95% CI: 3.35–8.02), whereas adding e-cigarettes to tobacco smoking did not enhance the likelihood of quitting tobacco and did not reduce tobacco cigarette consumption. E-smokers showed a minimal but significantly higher increase in self-rated health than other smokers. Non significant differences were found in self-reported serious adverse events (eleven overall).

**Conclusions:**

Adding e-cigarettes to tobacco smoking did not facilitate smoking cessation or reduction. If e-cigarette safety will be confirmed, however, the use of e-cigarettes alone may facilitate quitters remaining so.

**Registration Number:**

NCT01785537.

## Introduction

The electronic cigarette (e-cigarette) market has been growing sharply, reaching approximately $3 billion in 2013 worldwide [1]. Despite the broad public health relevance, the published evidence on e-cigarettes safety and efficacy in reducing traditional tobacco cigarette smoking is limited to two randomized trials [2, 3], two single-arm small trials [4–6], and six observational studies [7–12]. These studies mostly included smokers of both tobacco and e-cigarettes followed for 12 months or less, used various assessment methods, and reported controversial findings. Moreover, no direct comparisons between users of e-cigarettes only and smokers of tobacco cigarettes only are yet available [13].

In 2013 we started a 5-year study aimed at evaluating the long-term effects of e-cigarettes and to directly compare tobacco and e-cigarette exclusive smokers [14]. The final results are expected in 2019, but given the urgency of data to support policies on electronic smoking [1, 13, 15–17], we report here the main results of the 12-month follow-up.

## Methods

The protocol of this prospective cohort study has been reported elsewhere ([14], S1 File) and registered in Clinicaltrials.gov (NCT01785537). From June to November 2013 we recruited subjects through direct contact with general practitioners and e-cigarette shops, via internet advertisement and social networks. All participants were categorized to one of three natural and self-selected groups: tobacco smokers (if subjects had smoked ≥1 tobacco cigarette per day for the past 6 months); e-smokers (if subjects had been smokers of any type of e-cigarettes, inhaling ≥50 puffs weekly for the past 6 months); dual smokers (if subjects had smoked both tobacco and e-cigarettes within the same week for the past 6 months). Exclusion criteria were: age <30y and >75y; pregnancy or breastfeeding; illicit drug use, major depression, severe allergies, angina, and past episodes of smoking-related major diseases [14]. We originally recruited all volunteers and planned to check after two months from the start whether the distribution of smokers was largely unbalanced (given the much higher proportion of tobacco smokers only in the Italian population). At the 2-month check, we realized that the number of tobacco smokers only already exceeded the requested sample, and stopped their recruitment.

Data were collected through a structured questionnaire on smoking habits, previous and current diseases, lifestyle behaviour, and quality of life. The same questionnaire was administered through phone interview and/or by internet (www.ipazienti.it/fumo) after 12 months, and follow-up is scheduled to continue up to 60 months. Two investigators (MEF and LM) tested carbon monoxide levels in expired after breath (Smokerlyzer piCO+, Bedfont Scientific Ltd.) in a 25% random sample of those declaring tobacco smoking abstinence at the end of follow-up.

The primary outcome was the percentage of subjects reporting sustained (30 days) smoking abstinence from tobacco smoking at 12 months. Other outcomes were the proportion of quitters from all types of smoking (tobacco and e-cigarettes), the number of tobacco cigarettes smoked, self-reported health, and serious adverse events. Linear and logistic regressions, with region as cluster unit, were used to investigate potential predictors of continuous and categorical outcomes, respectively. Details on data analysis are reported in S2 File (web appendix).

The work was approved by the Ethics Committee of the University of Chieti (Record n. 6; 25-03-2013). All participants provided their written informed consent to participate in the study.

### Protocol violations

The study was originally planned to be funded, but the sponsor withdrew when the protocol was already approved and the study started. Therefore, the following deviations from the original protocol occurred due to funding limitations: (1) the sample was smaller than originally planned (500 subjects per group); (2) the 6-month follow-up interview was not made; (3) carbon monoxide levels were not tested in all quitters and non-relapsing e-smokers but only in a 25% random sample of them. Adverse events data will be checked for the residents in Abruzzo (46.1% of the sample) through data linkage with hospital and pharmaceutical administrative databases, but such datasets are typically provided with delay and could not be available at the first follow-up.

## Results

**Fig 1:**
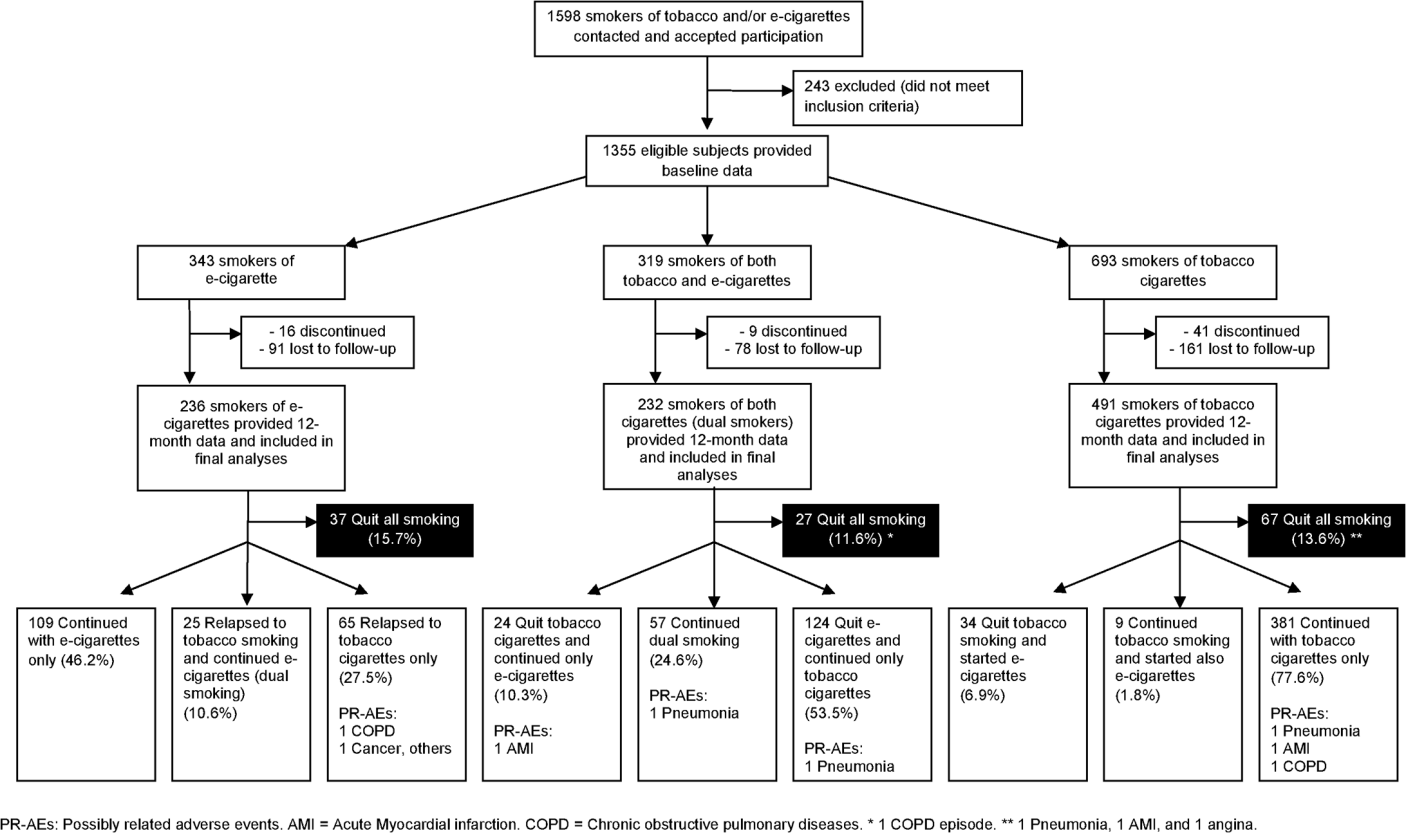
Flow of the participants and numbers of quitters and serious adverse events.

The flow of the participants is shown in Fig 1. Data at twelve months were available for 236 e-smokers, 491 tobacco smokers, and 232 dual smokers. Overall, the mean age was 44.5±11.6 years, and 55.9% were males. Few differences in baseline characteristics were found between participants completing the 12-month phase and withdrawals or lost to follow-up (29.2%; S1 Table), as well as among the three types of smokers (Table 1). All e-cigarette users were former tobacco smokers, since more than 20 years on average.

**Table 1:** Baseline characteristics of the subjects completing the 12-month follow-up.

	*Baseline smoking status*				
Variables	E-cigarettes only	Tobacco cigarettes only	Dual smoking	Overall	
	*(n = 236)*	*(n = 491)*	*(n = 232)*	*(N = 959)*	p*
Mean age in years (SD)	45.2 (10.7)	44.2 (11.9)	44.3 (12.0)	44.5 (11.6)	
Male gender, %	62.7	48.7	64.2	55.9	*, **
Mean BMI (SD)	24.7 (3.9)	24.4 (4.0)	24.8 (4.0)	24.6 (4.0)	
Married, %	60.5	54.8	55.7	56.4	
Employed, %	78.9	79.5	74.4	78.2	
*Educational level*, *%*					
- Elementary / Middle	21.9	21.9	22.1	22.4	
- High school	54.5	42.5	46.7	46.3	*
- Bachelor or higher	23.6	35.6	31.2	31.3	*
*Physical activity (69 missing)*					
- At work, %	18.9	20.3	15.7	18.9	
- Weekly hours at work, mean (SD)	23.8 (18.4)	26.7 (16.6)	22.9 (19.0)	25.3 (17.5)	
- At home, %	48.0	48.0	51.3	48.8	
- Weekly hours at home, mean (SD)	5.3 (4.7)	5.2 (5.5)	5.3 (4.5)	5.3 (5.1)	
*Alcohol use*					
Regular alcohol intake, %	20.0	29.4	27.4	26.6	*
Mean alcohol units daily (SD)	2.1 (1.2)	2.1 (1.6)	2.1 (1.0)	2.1 (1.4)	
*Cardiovascular risk and health*					
- Hypertension, %	13.6	11.6	9.9	11.7	
- Diabetes, %	4.2	3.3	4.3	3.8	
- Hypercholesterolemia, %	8.1	8.8	10.3	9.0	
- Self-reported health, mean (SD) ^€^	8.0 (1.3)	7.8 (1.3)	7.7 (1.2)	7.8 (1.3)	
- Low (<6) self-reported health ^€^, %	5.0	5.5	3.3	4.9	
*Smoking pattern*, *mean (SD)*					
- Years of tobacco smoking	21.4 (10.7)^¥^	22.3 (12.6)	25.2 (12.5)	22.9 (12.1)	**, ***
- N. tobacco cigarettes daily	—	14.1 (8.1)	14.9 (9.8)	14.4 (8.7)	
- Months of electronic smoking	8.8 (5.1)	—	8.4 (4.5)	8.6 (4.8)	
- N. e-cigarette daily puffs	162 (276)	—	96 (146)	130 (224)	***
- EC nicotine dose in mg	8.7 (5.2)	—	10.9 (5.6)	9.8 (5.5)	***
*E-cigarettes by nicotine dose*, *%*					
- No nicotine	12.8	—	5.6	9.3	***
- 3 to 8 mg	23.5	—	19.1	21.3	
- 9 mg	40.7	—	34.0	37.4	
- 10 to 24 mg	23.0		41.4	32.0	***
- Former tobacco smoking, %	100.0	100.0	100.0	100.0	
- Use of other tobacco products ^Ψ^, %	0.8	0.4	0.9	0.6	
- Use of other nicotine products ^Ω^, %	0.4	0.0	0.0	0.1	
*Reasons of e-cigarette smoking* ^φ^					
- Stop tobacco smoking, %	74.1	—	45.7	60.0	***
- Reduce tobacco smoking, %	16.9	—	56.5	36.5	***
- Indoor smoking, %	16.1	—	12.1	14.1	

^€^ EuroQol final question, ranging from 1 (feel very bad) to 10 (perfectly healthy). This item had 56 missing values.

^Ψ^ Cigars or tobacco chewing.

^Ω^ Nicotine patch or gums.

^φ^ More than one answer allowed.

^¥^ Years of former tobacco smoking for e-cigarette only smokers.

P<0.01 for the comparison

* Tobacco only vs electronic cigarettes only

** Tobacco only vs dual smoking

*** E-cigarettes only vs dual smoking.

**Fig 2:**
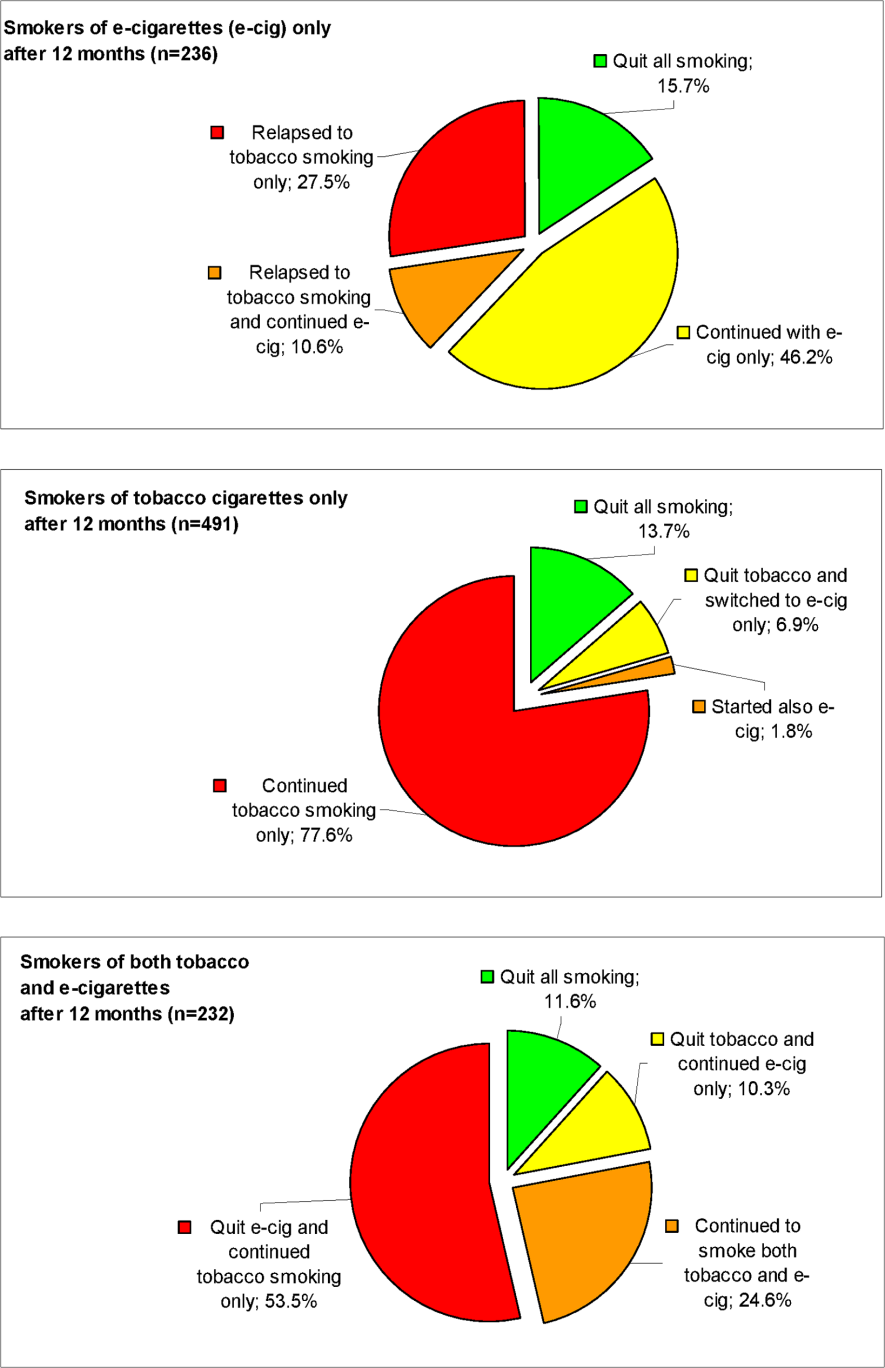
Smoking status after twelve months of follow-up, by smoking status at baseline.

After twelve months, 61.9% of the e-smokers were still abstinent from tobacco smoking; 20.6% of the tobacco smokers and 22.0% of the dual smokers achieved tobacco abstinence (Table 2). More than half (53.5%) of dual smokers abandoned e-cigarettes and continued to smoke only tobacco cigarettes (Fig 2). The proportion of subjects who quit all types of smoking (tobacco and e-cigarettes) did not significantly differ by baseline smoking status: 15.7%, 13.7% and 11.6% among electronic, tobacco and dual smokers, respectively (p>0.05). If analyzed with an Intention-To-Treat approach, which is however problematic given the large amount of switchers in the study, the above proportions of all-smoking quitters were 10.8%, 8.7% and 9.5%, respectively (all p>0.05). Only 41 (8.8%) e-smokers used nicotine-free e-cigarettes, and they were similar to nicotine e-smokers on both the rates of tobacco smoking relapse (43.9% vs 40.3%, respectively) and the rates of all-smoking cessation (14.6% vs 13.8%, respectively; all p>0.05).

**Table 2:** Main outcomes at twelve months.

	*Baseline smoking status*			
	E-cigarettes only	Tobacco cigarettes only	Dual smoking	
	(n = 236)	(n = 491)	(n = 232)	p*
**1. Smoking status**				
*Tobacco smoking*, *% (n)*				
- Tobacco smoking continuous abstinence or cessation, % (n)	61.9 (146)	20.6 (101)	22.0 (51)	*, ***
- Tobacco smoking (continued or relapsed)	38.1 (90)	79.4 (390)	78.0 (181)	*, ***
*All types of smoking*, *% (n)*				
- Quit all smoking (tobacco and e-cigarettes)	15.7 (37)	13.7 (67)	11.6 (27)	
- E-cigarettes	46.2 (109)	6.9 (34)	10.3 (24)	*, ***
- Both (mixed)	10.6 (25)	1.8 (9)	24.6 (57)	*, **, ***
- Tobacco cigarettes	27.5 (65)	77.6 (381)	53.5 (124)	*, **, ***
**2. Number of tobacco cigarettes—** *mean difference in the daily n*. *between 12 months and baseline (SD)*				
*Stratified by smoking status at baseline*	—	-3.1 (7.5)	-2.9 (11.6)	0.7
*Stratified by smoking status at 12 months*				
- Those who continued with the same type of smoking (no switch)	—	-1.5 (6.1)	-4.9 (10.8)	0.001
- Switched to tobacco cigarettes only (for those initially smoking both) or to both (for those initially smoking tobacco cigarettes only)	—	-6.2 (9.0)	+0.7 (9.9)	0.045
**3. Self-rated health** ^€^ *- mean difference between 12 months and baseline (SD)*				
*Stratified by smoking status at baseline*	+0.3 (1.5)	0.0 (1.5)	+0.1 (1.7)	*, ***
*Stratified by smoking status at 12 months*				
- Quit all smoking	+0.3 (1.4)	+0.2 (2.0)	+0.7 (1.7)	
- Smoking e-cigarettes only	+0.5 (1.3)	+0.9 (2.1)	+1.0 (1.4)	
- Smoking both tobacco and e-cigarettes	+0.6 (1.3)	-0.1 (1.7)	+0.1 (1.5)	
- Smoking tobacco cigarettes only	-0.4 (1.6)	-0.1 (1.4)	-0.1 (1.8)	
**4. Safety—** *possibly-related serious adverse events % (n)* ^*ψ*^				
*Stratified by smoking status at baseline*	0.9 (2)	1.2 (6)	1.7 (4)	
*Stratified by smoking status at 12 months*				
- Quit all smoking	0.0	4.5 (3)	3.7 (1)	
- Smoking e-cigarettes only	0.0	0.0	4.2 (1)	
- Smoking both tobacco and e-cigarettes	0.0	0.0	1.8 (1)	
- Smoking tobacco cigarettes only	3.1 (2)	0.8 (3)	0.8 (1)	

SD = Standard deviation.

P<0.01 for the comparison

* Tobacco only vs electronic cigarettes only

** Tobacco only vs both tobacco and electronic cigarettes (dual smoking)

*** E-cigarettes only vs both tobacco and electronic cigarettes (dual smoking).

^€^ EuroQol final item, ranging from 1 (feel very bad) to 10 (perfectly healthy).

^ψ^ Chronic obstructive pulmonary diseases, stroke, heart failure, myocardial infarction, angina, pneumonia, cancer of: larynx or oral cavity, lung, stomach, pancreas, cervix, kidney, bladder, myeloid leukaemia.

Of the 154 subjects initially declaring tobacco smoking cessation during follow-up, and of the 147 e-smokers declaring prolonged tobacco abstinence, 38 and 36 underwent a test to detect exhaled CO levels, respectively. CO levels suggestive of tobacco smoking (>7 ppm) [18] were found in only three subjects (two tobacco smokers and one e-smoker), who admitted the error and were accordingly re-classified.

The results on the change in tobacco cigarette consumption from baseline were controversial. On one side, the percentage of subjects who reduced the number of tobacco cigarettes smoked per day by 50% or more from baseline was similar among dual and tobacco only smokers (29.2% and 29.4%, respectively). On the other side, the mean daily consumption of tobacco cigarette varied widely according to 12-month smoking status (Table 2): among dual smokers, those who continued to smoke both tobacco and e-cigarettes reduced their mean consumption by ≈5 cigarettes per day (from 14.0 to 9.3 cigarettes/daily; p = 0.002; **S2 Table**), whereas those who quit e-cigarettes and continued only tobacco smoking slightly increased their daily cigarette number by 0.7 (from 15.4 to 16.1 cigarettes/daily; p = 0.5; S2 Table). Symmetrically, the reduction in daily cigarette number was higher among the tobacco smokers who started smoking also e-cigarettes (from 19.0 to 12.8; -6.2±9.0 cigarettes/daily; p = 0.07; Table 2 and S2 Table) rather than those who continued with tobacco cigarettes only (from 14.3 to 12.8; -1.5±6.1 cigarettes/daily; p<0.001; Table 2 and S2 Table).

Although significant, a minimal increase from baseline in self-rated health score was observed among e-smokers only (+0.3±1.5; p = 0.013), while it remained stable in both tobacco and mixed groups (Table 2 and S2 Table). Non-significant increases in self-rated health were observed among mixed smokers who quit, and tobacco smokers who switched to e-cigarettes only.

Overall, 11 possibly-related (4 pneumonia, 4 COPD, 3 myocardial infarction, 1 angina) and 1 unrelated (brain cancer) serious adverse events were reported by participants: 2 among the e-smokers (both switched to tobacco smoking during follow-up); 6 among tobacco smokers (3 quit all smoking); 4 among mixed smokers (all switched smoking but one). Of the twelve subjects reporting a serious adverse event, at baseline 4 were hypertensive and 1 diabetic (distributed across smoking groups). No deaths occurred.

Multivariate analyses confirmed univariate results (Table 3): even when several potential confounders were adjusted for, tobacco smoking abstinence or cessation remained significantly more likely among e-smokers (adjusted OR 5.19; 95% CI: 3.35–8.02), whereas adding e-cigarettes to tobacco smoking did not enhance the likelihood of quitting tobacco and did not reduce tobacco cigarette consumption. E-smokers showed a significantly higher increase in self-rated health than other smokers, while the likelihood of quitting all types of smoking did not significantly vary by smoking status.

**Table 3:** Smoking abstinence and cessation, difference in the number of daily cigarette smoked and self-reported health: results of the multivariate analyses.

	**Adjusted OR**	**p**	**Crude OR**	**p**
**Outcomes at 12 month**	**(95% CI)**		**(95% CI)**	
*Tobacco smoking continuous abstinence or cessation* ^*A*^				
Tobacco cigarettes only (ref. cat.)	1	—	1	—
E-cigarettes only	5.19 (3.35; 8.02)	<0.001	5.03 (3.41; 7.40)	<0.001
Both tobacco and e-cigarettes	0.83 (0.53; 1.29)	0.4	0.93 (0.62; 1.40)	0.7
*All smoking cessation* ^*A*^				
Tobacco cigarettes only (ref. cat.)	1	—	1	—
E-cigarettes only	1.25 (0.77; 2.04)	0.4	1.18 (0.76; 1.82)	0.5
Both tobacco and e-cigarettes	0.70 (0.41; 1.19)	0.2	0.83 (0.52; 1.34)	0.5
	**Adjusted coefficient**		**Raw coefficient**	
	**(95% CI)**		**(95% CI)**	
*Difference in the daily n*. *or tobacco cigarettes from 12 months to baseline* ^*B*^				
Tobacco cigarettes only (ref. cat.)	0	—	0	—
Both tobacco and e-cigarettes	0.60 (-0.70; 1.91)	0.4	0.28 (-1.23; 1.78)	0.7
*Difference in the self-reported health score from 12 months to baseline* ^*C*^				
Tobacco cigarettes only (ref. cat.)	0	—	0	—
E-cigarettes only	0.31 (0.04; 0.59)	0.026	0.25 (0.00; 0.50)	0.047
Both tobacco and e-cigarettes	0.14 (-0.13; 0.40)	0.3	0.13 (-0.13; 0.39)	0.3

OR = Odds Ratio. CI = Confidence Interval.

^A^ Random-effect logistic regression with region as the cluster level, adjusting for the following baseline characteristics: age, gender, BMI, marital status, educational level, occupation, alcohol use, hypertension, hypercholesterolemia, diabetes, self-reported health, years of tobacco smoking (former smoking for e-cigarette users), n. of tobacco cigarettes smoked per day (or puffs per day for e-cigarette only smokers). 903 subjects were included in the final model due to 56 missing items in the self-reported health item at baseline.

^B^ E-cigarette only smokers were not included. Random-effect linear regression with region as the cluster level, adjusting for the following baseline characteristics: age, gender, BMI, marital status, educational level, occupation, alcohol use, hypertension, hypercholesterolemia, diabetes, self-reported health, years of tobacco smoking, n. of tobacco cigarettes smoked per day. 685 subjects were included in the final model due to 38 missing items in the self-reported health item at baseline.

^C^ Random-effect linear regression with region as the cluster level, adjusting for the following baseline characteristics: age, gender, BMI, marital status, educational level, occupation, alcohol use, hypertension, hypercholesterolemia, diabetes, self-reported health, years of tobacco smoking (former smoking for e-cigarette users), n. of tobacco cigarettes smoked per day (or puffs per day for e-cigarette only smokers). 874 subjects were included in the final model due to 56 missing items in the self-reported health item at baseline and 29 missing items in the self-reported health at twelve months.

The only other significant predictors of tobacco and all-smoking abstinence were age (adjusted ORs 0.90 and 0.96, respectively, for each 5-year increase; both p<0.03) and BMI (for tobacco cessation only; OR 1.06 and p = 0.004 for each BMI unit increase). Older age was also associated with a decrease in self-rated health during follow-up (coeff. -0.06 for each 5-year increase; p = 0.027), as it was diabetes mellitus (coeff. -0.82; p = 0.010). Only the daily number of tobacco cigarettes at baseline was significantly, inversely associated with their reduction during follow-up (coeff. 0.56 for each cigarette increase; p<0.001).

## Discussion

E-cigarettes are a controversial issue. Some experts suggested that e-cigarette use should be restricted or banned [19] because of their potential to increase tobacco cigarette consumption (reducing motivation to completely quit, acting as a gateway to smoking for non-smokers, or increasing smoking social acceptability) [19–25], and because concerns have been raised on the potential harms from propylene glycol [19, 24], cartridge manufacturing and content quality (as refills may contain toxins and cause nicotine poisoning) [16, 19, 24], and on the potential risks from second-hand electronic smoking in indoor environments [15, 19]. On the contrary, other experts claimed that e-cigarettes may be the centrepiece of a harm reduction strategy (being more attractive and cheap than other nicotine replacement therapies and facilitating smoking cessation, reduction or abstinence), that there is no evidence of undesirable uptake from non-smokers, that e-cigarettes pose only a small fraction of the risks of tobacco cigarettes (as tested liquids and aerosols contain negligible concentrations of toxicants and carcinogens, and use of nicotine without tobacco toxicants poses little risks for most of the population) [16, 25–28]. They therefore suggest that no regulations other than quality controls should be introduced, in order to encourage smokers to switch to a safer form of addiction [16, 27]. At least, there is universal consensus that current evidence is scarce and long-term data on e-cigarette safety and efficacy are urgently needed [16, 19–23, 25, 29, 30].

This is the first study directly comparing smokers of tobacco cigarettes only with users of e-cigarettes only, and aimed at providing safety data on the largest sample of e-cigarette users for the longest time. On one side, the use of e-cigarettes in addition to tobacco (dual smoking) did not seem to improve neither smoking cessation nor reduction: as compared with tobacco only smokers, dual smokers showed a similar quitting rate, no difference in self-rated health, and a non-significant reduction in the number of cigarettes smoked daily. On the other side, no safety concerns raised during the study, although the limitations in adverse events recording prevent us to draw any conclusions. The users of e-cigarettes only showed a minimal though significant increase in self-rated health and, most importantly, they showed a 1-year relapse rate which is relatively low (38.1%) when compared with real-life rates (60% to 90%) of tobacco-cigarette smokers [31]. Indeed, such a low relapse rate could be partially explained by the fact that, in this sample, the users of e-cigarettes only did quit tobacco smoking already when the study started (since 8 months on average). Thus, the high proportion of e-cigarette users who did not relapse (that is, remained quitters) cannot be interpreted tout-court as a smoking cessation rate. Given this, if e-cigarettes will be proved safer than tobacco cigarettes, this finding may still support the utility of e-cigarettes as a tool to help quitters remaining so. Clearly, its interpretation would be drastically different if e-cigarettes would prove to be equally or even less safe than tobacco cigarettes: although unlikely, in such a case the high adherence to e-cigarette use would be an extremely negative finding, eventually supporting more restrictive regulations and/or a complete ban.

Current data on serious adverse events in e-cigarette users are limited. Only three studies evaluated such an outcome on healthy subjects [2, 3, 6]. Moreover, in one study the intervention with e-cigarettes lasted 3 months [3], and in the other two studies those who continued e-cigarette use for six months were, in total, 27 [5] and 95 (one third of the sample excluded, protocol violation n. 1) [2]. Thus, although the methodology of the above studies was rigorous, so far the entire evidence on e-cigarette safety at 6 months is limited to 122 subjects, most of whom were also smoking tobacco for most of the follow-up. In the present study, data are provided on 134 baseline e-smokers only and 81 dual smokers who continued to use e-cigarettes for twelve months. We observed a low rate of serious adverse events, with no differences by smoking status. Safety data were self-reported and it was not possible to establish whether such events may be related to smoking. Also, considering that also our participants were past tobacco smokers, even among the smokers of e-cigarettes only some more reliable information will be available exclusively in the next years of follow-up, when also administrative data will be available to check self-reported data. Therefore, although the present results agree with previous studies [2, 3, 5] and FDA Center for Tobacco Products data [32] suggesting no increase in health risks with e-cigarette use, they do not allow to draw any firm conclusion and more research is strongly needed on electronic smoking safety, which remains the most important issue from a public health perspective.

Our findings on smoking cessation and reduction rates of tobacco smokers are comparable to those by Choi et al. [9] and Adkinson et al. [8], slightly higher than those reported by Grana et al. among smokers unwilling to quit [10], and lower than the highest 31.3% quit rate reported by Vickerman et al. among quit-line callers [11]. Smoking reduction outcomes in our dual smokers were also similar to most reported data [5, 6, 8], and higher than those reported by Caponnetto et al. among smokers of ≥10 cigarettes/day unwilling to quit [3]. Our smoking cessation rate in dual smokers was however comparable or lower than that from three studies only [7, 11, 12] and higher than several other studies [3, 6, 8–10]. As a potential explanation, three of such studies may have had less motivated samples (smokers unwilling to quit) [3, 6, 10], and another included countries with an e-cigarette ban and was made during previous years (data collected from 2008 to 2011), thus the motivation and/or the possibility to switch to e-cigarette only might have reduced [8]. Our relapse rate among the users of e-cigarettes only more than doubled the estimate by Etter et al. (whose sample, however, may have been highly motivated as recruitment started from smoking cessation websites [7]), but it was much lower than the ≅94% relapse rate documented in the RCT by Bullen et al. [2]. However, such data cannot be compared directly, as our observational design included e-cigarette users that were already spontaneous quitters since eight months on average, smoked any type of e-cigarette rather than those provided by investigators, and were not selected as smokers of ≥10 cigarettes daily. In any case, such a discrepancy indicates that our low relapse rate among e-smokers should only be interpreted as the abstinence achieved by people who already quit, not as the smoking cessation rate that e-cigarette (alone) might have if used as an intervention among smokers who did not quit.

This study has some limitations. First, smoking cessation was mostly self-reported, as we were able to test carbon monoxide levels only in 25% of the sample in abstinence from tobacco smoking. In such a sample, however, only 4% of the quitters (n = 3) had to be reclassified. Second, we had no follow-up data for almost 30% of baseline participants, a loss rate lower than most previous prospective studies [3, 6, 7, 10–12] but still prone to bias. However, non-responders were similar to responders for most variables. In any case, those accepting to participate to the study and providing follow-up data are likely to be more motivated, which may partially explain the high smoking cessation rate even when e-cigarette switchers are excluded (13.7%). As in previous Italian studies [3, 6], even if cessation rates are likely to be overestimated with respect to the Italian average population (www.istat.it), there are no reasons to believe that this should bias the differences by smoking status. Third, like all previous observational studies we included all types of e-cigarettes to approach real-life conditions, but different e-cigarette models with various nicotine doses might lead to diverse results. However, in this study the rates of tobacco abstinence and all-smoking cessation of the users of nicotine-free e-cigarettes were similar to those of nicotine e-cigarette users. In agreement with previous data [2, 3, 5], such finding deserves further investigation in order to better understand the role of rituals such as handling and manipulation in determining smoking dependence [3].

### Conclusions

In conclusion, during the 12-month follow-up no serious safety concerns emerged among the largest sample of e-cigarette users examined so far. Such data, however, must be considered preliminary. Adding e-cigarettes to tobacco smoking did not facilitate smoking cessation or reduction, as dual smokers and tobacco only smokers showed similar outcomes. In contrast, most exclusive users of e-cigarettes were able to maintain smoking abstinence at twelve months. If e-cigarette safety will be confirmed in long-term evaluations including the present, they may be a promising tool to help quitters remain so.

## Supporting Information

S1 File Study protocol. (PDF)

S2 File Outcome variables and data analysis. (DOC)

S1 Table Characteristics of the subjects completing the 12-month follow-up vs withdrawals or lost-to-follow-up subjects. (DOC)

S2 Table Secondary outcomes. (DOC)
