# Supplementary material for: Electronic Cigarettes Efficacy and Safety at 12 Months: Cohort Study
Source: PLoS One. 2015 Jun 10;10(6):e0129443. doi: 10.1371/journal.pone.0129443 (PMC4464650; doi:10.1371/journal.pone.0129443)
Supplement: S1 File — (PDF) [file pone.0129443.s001.pdf]

BMC Public Health

STUDY PROTOCOL Open Access

Multicentric cohort study on the long-term efficacy and safety of electronic cigarettes: study design and methodology

Lamberto Manzoli[1*], Carlo La Vecchia[2,3], Maria Elena Flacco[1], Lorenzo Capasso[1], Valentina Simonetti[4], Stefania Boccia[5], Angela Di Baldassarre[1], Paolo Villari[6], Andrea Mezzetti[7] and Giancarlo Cicolini[4]

Abstract

Background: While electronic cigarettes are forbidden in several countries, their sales are exploding in many others. Although e-cigarettes have been proposed as long-term substitutes for traditional smoking or as a tool for smoking cessation, very scarce data are available on their efficacy and safety.

We describe the protocol of a 5-year multicentric prospective study aimed to evaluate short- and long-term adherence to e-cigarette smoking and the efficacy of e-cigarettes in reducing and/or quitting traditional cigarette smoking. The study will also compare the health effects of electronic vs traditional vs mixed cigarette smoking.

Methods/design: From June to December 2013, we will enroll adult smokers of: (EC) e-cigarettes (self-reported inhaling ≥ 50 puffs per week since ≥ 6 months); (TC) traditional cigarettes (≥ 1 per day since ≥ 6 m); (Mixed) both electronic and traditional cigarettes (≥1 per day since ≥ 6 m). Eligible subjects will be requested participation through newspaper advertisements and direct contact at the shops. Each subject will have to compile a structured questionnaire at enrolment and after 6, 12, 24, 36 and 60 months. The level of carbon monoxide in expired after breath will be evaluated in all subjects declaring no traditional cigarette smoking in any follow-up phase, using portable carbon monoxide analyzers. The primary outcomes are traditional smoking cessation rates and number of smoked cigarettes. Secondary outcomes include adherence to e-cigarettes, self-reported adverse events, quality of life, and time to hospital admission for one among cardiovascular diseases, chronic obstructive pulmonary diseases, cancer of the lung, esophagus, larynx, oral cavity, bladder, pancreas, kidney, stomach, cervix, and myeloid leukemia. Admissions will be checked using official discharge data of the Abruzzo Region. A minimum of 500 subjects in each group will be enrolled, for a total of 1500 participants. Cox proportional hazards analysis will be used to calculate adjusted relative hazards of smoking cessation by each variable.

Discussion: Data on long-term efficacy and safety of e-cigarettes will be of utmost importance to form the basis for guidelines and regulatory decisions on e-cigarettes.

Trial registration: The protocol has been registered (NCT01785537) and approved by the Ethics Committee of the University of Chieti (Record n. 6; 25-03-2013).

Keywords: Electronic cigarette, Cigarette smoking, Efficacy, Safety, Cohort study, Italy

* Correspondence: lmanzoli@post.harvard.edu
[1]Department of Medicine and Aging Sciences, University of Chieti, via dei Vestini 5, 66013 Chieti, Italy
Full list of author information is available at the end of the article

BioMed Central

© 2013 Manzoli et al.; licensee BioMed Central Ltd. This is an Open Access article distributed under the terms of the Creative Commons Attribution License (http://creativecommons.org/licenses/by/2.0), which permits unrestricted use, distribution, and reproduction in any medium, provided the original work is properly cited.

Background

The electronic cigarette (e-cigarette) market has been exploding over the past few years, reaching an estimated $500 millions during 2012 in USA; an expected $1 billions by the end of 2013 [1,2]; and achieving similar growth in some European countries [3,4]. Indeed, people report buying e-cigarettes to help quit smoking, to reduce cigarette consumption, to relieve tobacco withdrawal symptoms due to workplace smoking restrictions and to continue to have a 'smoking' experience but with reduced health risks [5]. As a matter of fact, e-cigarette is becoming a social phenomenon of global proportion [6,7], which calls for research, legislation and product development [8,9].

Although the product has been proposed as a long-term substitute for traditional smoking or as a tool for smoking cessation [10,11], some authorities are opposing the recreational use of nicotine because there are concerns about both safety and efficacy, and about the potential risk of e-cigarettes to play as a promoter of smoking for never or ex-smokers [10,12-14]. In fact, the sale of e-cigarettes market is subject to limitations in many countries (i.e. USA and Germany) and is forbidden in several others (i.e. China, Brazil) [14].

To date, twelve studies were started evaluating the acceptability, safety or efficacy of e-cigarettes containing nicotine [5,15-25]; seven were completed [16-21,24], and only four have been published [16,17,24,26]. Although the results are encouraging, they are based upon a total sample of <300 subjects, who were followed for no more than 6 months. No data are available on long-term efficacy and safety and, most importantly, no ongoing study has been designed to collect data on hard outcomes (smoking-related cancer, major cardiovascular events and chronic obstructive pulmonary disease – COPD) over a long follow-up period [11,24].

The main aims of the study are to evaluate for the first time the long-term adherence to e-cigarette smoking and the efficacy of e-cigarettes in reducing and/or quitting traditional cigarette smoking. As secondary aims, the study will also evaluate the long-term safety (in terms of smoking-related serious diseases requiring hospitalization) of e-cigarette smoking, comparing its health effects with those of traditional cigarette smoking and mixed electronic and traditional cigarette smoking. We will evaluate over a 5-year follow-up the number of smoking-related hospitalizations, the self-reported quality of life, and the reported adverse events according to current and past smoking habit.

The present paper describes the research protocol of the project, which is currently on course (NCT01785537).

Methods/design

Study design and population

This is an observational, prospective cohort study. None of the participants will receive an indication about which product to smoke. In fact, all participants will receive structured flyers reporting the main harms of cigarette smoking.

An individual will be considered for participation if:

– resident in Italy;
– aged between 30 and 75 years;
– smoker of e-cigarettes (inhaling at least 50 puffs per week) since six or more months (EC Group);
– smoker of at least one traditional cigarette per day since six or more months (TC Group);
– smoker of both electronic and traditional cigarettes (at least one per day) since six or more months (Mixed Group).

Exclusion criteria will be:

– illicit drug use,
– breastfeeding or pregnancy,
– major depression or other psychiatric conditions,
– severe allergies,
– active antihypertensive medication,
– angina pectoris,
– past episodes of selected smoking-related major diseases (myocardial infarction, stroke/TIA, congestive heart failure, COPD, cancer of the lung, esophagus, larynx, oral cavity, bladder, pancreas, kidney, stomach, cervix, and myeloid leukemia).

Potentially eligible subjects will be requested participation through newspaper and internet advertisements, through direct contact with an investigator at the shops, finally through direct contact with some of the general practitioners of the Abruzzo Section of the Italian Scientific Society of General Medicine (SIMG) who voluntarily participate to the project. Specific agreements will be established with the sellers to permit the presence of an investigator during conventional working hours. Inclusion and exclusion criteria will be self-reported and further verified by the investigator in case of participation.

The enrolment period will last from June to December 2013. Each subject will be requested to compile a structured questionnaire at the time of enrolment at after 6, 12, 24, 36 and 60 months (and eventually, in the case of an expansion of the follow-up, at 84 and 120 months). After the first contact, the follow-up questionnaires will be compiled through a phone interview or through a specifically created website (http://www.ipazienti/fumo), with private password. To reduce potential misclassification, the level of carbon monoxide in expired after

breath will be evaluated in all subjects declaring no traditional cigarette smoking in any phase of the follow-up, using portable carbon monoxide analyzers (Smokerlyzer ® piCO + ™, Bedfont Scientific Ltd.). Such an assessment will be made by the investigators, after specific periodical calibration, directly at the home of the participant or at the office of his/her general practitioner. Among the methods to monitor self-reported smoking cessation, also the assessment of cotinine in saliva can be made using portable and relatively economical devices, and such test showed a slightly higher sensitivity/specificity than tests based upon exhaled CO [27]. However, we were not able to find a seller of cotinine portable tests on saliva, and the measurement of breath CO still showed high levels of sensitivity (>90% [27,28]).

Each participant will have to sign a written informed consent. The study protocol has been registered in Clinicaltrials.gov (NCT01785537) and approved by the Ethics Committee of the University of Chieti (Record n. 6; 25-03-2013).

Participants will be enrolled at various study sites within the Abruzzo and Lazio Region using the same standardized procedures.

All the subjects that will be excluded because of past episodes of selected smoking-related major diseases will be requested to participate into a nested case–control pilot study, in which we will collect hospital admission data retrospectively and compare the rates of smoking-related diseases according to the present and past smoking habits.

Interventions and groups

This is a non-interventional observational study. The participants will be divided into three cohorts/groups according to their self-determined smoking pattern:

– Group EC - smokers of e-cigarettes only (who are not smoking traditional cigarettes). This group will be further split in the analysis: never or former smokers of traditional cigarettes; smokers of e-cigarettes with or without nicotine.
– Group TC - smokers of traditional cigarettes only. This group will be further split in the analysis: recent and older smokers.
– Mixed Group - smokers of both traditional and electronic cigarettes. This group will be further split in the analysis: mixed smokers who quit and who did not quit traditional cigarette smoking during follow-up.

Questionnaire

The structured questionnaire is available as online supplementary material and contains questions on demographic data (gender, age, height and weight, educational level, marital status, occupation), previous diseases which are and are not among the reasons for the exclusion, smoking habits, smoking related diseases which did or did not require hospitalizations (only in follow-up assessments), lifestyle behavior (physical activity, alcohol use) and quality of life. The latter issue will be investigated using the Italian version of the validated EuroQol EQ-D5L questionnaire [29]. The questionnaire will be kept as short as possible to allow phone interviews and will be initially tested on a sample of 50 participants to assess acceptability, exclude potentially redundant or include potentially useful questions.

Primary outcomes

1. Traditional smoking cessation: percentage of subjects that were current (in TC and Mixed groups) or former (in EC group) smokers reporting sustained smoking abstinence from traditional cigarette smoking at 6, 12, 24, 36 and 60 months. Smoking abstinence is defined as complete abstinence from tobacco smoking (not even a puff) for the 30 days period prior to the visit.
2. Decrease in the number of traditional cigarettes smoked: average difference in self-reported number of traditional cigarettes smoked per day at baseline and 6, 12, 24, 36 and 60 months in the groups TC and Mixed.

Secondary outcomes

1. Adherence to e-cigarette smoking: number of months of continued e-cigarette smoking in groups EC and Mixed.
2. Traditional and electronic smoking (overall smoking) cessation: percentage of subjects in all groups reporting sustained smoking abstinence from both traditional and electronic cigarette smoking at 6, 12, 24, 36 and 60 months. Smoking abstinence is defined as complete abstinence from tobacco or electronic smoking (not even a puff) for the 30 days period prior to the visit.
3. Time to hospital admission for one of the followings: cardiovascular diseases, chronic obstructive pulmonary diseases, cancer of the lung, esophagus, larynx, oral cavity, bladder, pancreas, kidney, stomach, cervix, and myeloid leukemia. Repeated admissions of the same subject will be counted once and the subject will be censored at the date of the first admission.
4. Rate of subjects with smoking-related hospitalizations: percentage at 1, 2, 3 and 5 years of subjects who had a hospital admission for one of the followings: cardiovascular diseases, chronic

obstructive pulmonary diseases, cancer of the lung, esophagus, larynx, oral cavity, bladder, pancreas, kidney, stomach, cervix, and myeloid leukemia. Repeated admissions of the same subject will be counted once.

5. Number of smoking-related hospitalizations: mean number at 1, 2, 3 and 5 years of hospital admissions for one of the followings: cardiovascular diseases, chronic obstructive pulmonary diseases, cancer of the lung, esophagus, larynx, oral cavity, bladder, pancreas, kidney, stomach, cervix, and myeloid leukemia. Each admissions of the same subject will be counted.
6. Number of hospitalizations for cardiovascular diseases: mean number at 3 and 5 years of hospital admissions for cardiovascular diseases. Each admissions of the same subject will be counted.
7. Number of hospitalizations for smoking-related cancers: mean number at 3 and 5 years of hospital admissions for cancer of the lung, esophagus, larynx, oral cavity, bladder, pancreas, kidney, stomach, cervix, and myeloid leukemia. Each admissions of the same subject will be counted.
8. Self-reported quality of life: average quality of life according to EuroQoL EQ-5DL at 6, 12, 24 and 36 months. The analysis of quality of life will follow the technical specifications recommended by the EuroQol group [29].
9. Reported side effects at 6, 12, 24, 36 and 60 months.

All outcomes will be self-reported; primary outcome 1 and secondary outcome 2 will be checked using portable CO analyzers, and secondary outcomes 3–7 will be checked using hospital discharge abstracts through a linkage to fiscal codes (for the residents in Abruzzo only). The permission will be granted from each participants and hospitalization data will be provided by the Regional Healthcare Agency of Abruzzo. The admissions for each of the conditions included in the composite outcomes will be defined as follows, based upon the ICD-9-CM principal diagnosis codes in the Italian hospital discharge abstracts (SDO):

– COPD or asthma - 490, 490.0, 491.1, 491.2, 491.21, 491.8, 491.9, 492.0, 492.8, 494, 494.0, 494.1, 496, 493.00, 493.01, 493.02, 493.10, 493.11, 493.12, 493.20, 493.21, 493.22, 493.81, 493.82, 493.90, 493.91, 493.92 [30]
– Myocardial infarction - 410.0-410.9
– Congestive heart failure - 428.0-428.9
– Transitory cerebrovascular ischemia - 435.0-435.9
– Stroke - 433.0-434.9
– Cancer of the lung - 162.0-162.9
– Cancer of the esophagus - 150.0-150.9
– Cancer of the larynx - 161.0-161.9
– Cancer of the oral cavity - 140.0-149.9
– Cancer of the bladder - 188.0-188.9
– Cancer of the pancreas - 157.0-157.9
– Cancer of the kidney - 189.0
– Cancer of the, stomach - 151.0-151.9
– Cancer of the cervix - 180.0-180.9
– Myeloid leukemia - 205.0-205.9

Sample size estimation

The primary analyses will compare: (A) the primary outcome 1 (rate of traditional smoking sustained cessation) across the groups TC, Mixed and EC (only the subjects that were former traditional smokers); (B) the difference in the number of traditional cigarettes smoked per day between baseline and end of follow-up in groups TC and Mixed.

As regards primary analysis 1, conservatively assuming a 5-year cessation rate of 20% in the TC group, and 30% in the Mixed group (EC group most probably having a higher cessation rate), we would need 313 subjects per group to achieve a power of 80%. Given the need of multivariate modeling and an expected 20% rate of withdrawals/dropouts, 500 subjects would be required in each group.

Concerning primary analysis 2, assuming a 5-year reduction in the number of smoked traditional cigarettes of 2 ± 6 and 4 ± 6 in the Mixed group, with the same above parameters (alpha error = 0.05; beta-error = 0.20; dropout/withdrawals = 20%), we would only require 240 subjects per group.

We will thus conservatively enroll a minimum of 500 subjects in each group (EC, TC and Mixed), for a total of 1500 participants.

Data analysis

All data will be collected and initially handled by the Coordinating unit, which is responsible for privacy regulations and maintains the property.

At each time-point, the differences in the rates of traditional smoking cessation (or other categorical outcomes such as adverse events) across groups will be first examined using Fisher's exact test. Cox proportional hazards analysis will be used to calculate the adjusted relative hazards of traditional smoking cessation (and smoking-related hospitalization) by each variable. Stochastic level of entry into the model will be set at 0.10, with age, gender, and smoking groups forced to entry, and multicollinearity, interactions with time to event and higher-power terms will be explored for all variables in the final model. A minimum events-to-variable ratio of 10 will be maintained in multivariate modeling to avoid overfitting, and Schoenfeld's test will be performed to check the validity of proportional hazards assumption. Kaplan-Meier survival analysis will

also be used to examine the association between hospitalization and smoking group. The validity of constant incidence ratios over the follow-up will be checked using Nelson-Aalen cumulative hazard estimates.

Multiple linear regression will be used to investigate the predictors of the difference in the number of traditional cigarettes smoked between baseline and each time-point of follow-up. Additional multivariate models such as mixed-effect logistic models will be explored for both the primary and secondary outcomes to account for repeated observations and the potential distortions of the variability in the exposure to traditional cigarette smoking by the participants in groups EC and Mixed (for which also stratification will be considered) [31]. In all mixed models, the cluster variables will be participant's id, nicotine level of the electronic and traditional cigarette and exposure to past traditional cigarette smoking. We will assume and independent correlation structure for all clusters, but we will also repeat all models setting an exchangeable correlation structure.

The prevalence of e-cigarette smoking compliance (secondary outcome n. 1) will similarly be derived at 6, 12, 24, 36 and 60 months. However, with the exception of the 6-months cut-off (which will probably include the vast majority of participants), the crude estimates of adherence for the following time-points would most probably be underestimated, as several participants who are still adherent will be excluded because they will not be followed up to the cut-off point. Therefore, adjusted estimates of adherence for each follow-up point will be calculated using the Nelson-Aalen method for censoring, which assigns censored observations according to the corresponding period of failure incidence [32].

A p-value of <0.05 will be considered significant for all analyses, which will be performed using STATA statistical software, version 11.1 (Stata Corporation, College Station, TX, USA, 2007).

Timetable, participating units and funding

We will start in June, 2013, and the enrolment period will end in December 31, 2013. The preliminary data on outcomes which do not require a first checking through hospital discharge abstracts will thus be available by the end of 2014. A first publication is consequently scheduled for the second semester of 2014, and it will report also the results of the pilot nested case–control study on the subjects that accepted to participate but were excluded from the prospective cohort study because of past episodes of major diseases.

Hospital discharge abstracts are typically available in the Abruzzo region four months after the end of the year. Thus, we plan to obtain the first data on hospitalizations by May 2014, and to finish the data collection by May 2019. The final results of the study will therefore be available at the end of the first semester 2019. Eventually, in case of funds availability and approval by the Ethics Committee, the follow-up could be extended up to ten years.

The following units will participate in the project:

- Department of Medicine and Aging Sciences, University of Chieti (Coordinating unit and sponsor)
- Healthcare Professions Unit, Local Health Authority of Lanciano-Vasto-Chieti (recruitment and data collection)
- University of Turin (recruitment and data collection)
- University Sapienza of Rome (study design and analyses)
- University “Catholic of the Sacred Heart” of Rome (statistical analyses and publication support)

Other institutions and sponsors could be involved during the course of the study. In particular, additional sponsors will be actively searched.

Discussion

While electronic cigarettes are forbidden in several countries, their sales are exploding in many others [1-3,14]. Data from multicenter prospective studies evaluating the long-term health effects of e-cigarettes and their utility to induce traditional smoking reduction or cessation are strongly needed and will be of utmost importance to form the basis for guidelines and regulatory decisions on e-cigarettes, which may prove to be the most promising solution for the reduction of the health damages associated with conventional smoking [10,11].

Competing interests

The authors declare they have no competing interests.

Authors' contributions

All the authors contributed to the conception, design and are currently carrying out the study. LC, MEF and LM wrote the present manuscript. All authors read and approved the final manuscript.

Acknowledgments

The study is funded using internal research funds of the University of Chieti (ex 60% MIUR funds). The authors are grateful to Dr. Lucio Zinni, Gabriella Salladini, and all the colleagues of the Abruzzo Section of the Italian Scientific Society of General Medicine (SIMG) for their voluntary support to the enrollment of subjects.

Author details

[1]Department of Medicine and Aging Sciences, University of Chieti, via dei Vestini 5, 66013 Chieti, Italy. [2]IRCCS Istituto di Ricerche Farmacologiche “Mario Negri”, Via la Masa 19 20156 Milan, Italy. [3]Department of Clinical Sciences and Community Health, University of Milan, Via Commenda 9/12, 20156 Milan, Italy. [4]Local Health Authority of Lanciano-Vasto-Chieti, Via Martiri Lancianesi, 66100 Chieti, Italy. [5]University “Catholic of the Sacred Heart” of Rome, Largo Francesco Vito, 1-00168 Rome, Italy. [6]University Sapienza of Rome, Viale Regina Elena 324, 00161 Rome, Italy. [7]Clinical Research Center, Ce.S.I., University “G. d'Annunzio” Foundation, Via Colle dell'Ara, 66013 Chieti, Italy.

Received: 27 May 2013 Accepted: 20 September 2013
Published: 24 September 2013

References

1. UBS WF, Tobacco Vapor Electronic Cigarette Association: *Electronic cigarette statistics*; 2013. Available at http://www.statisticbrain.com/electronic-cigarette-statistics/.
2. UBS Investment Research: *U.S. Tobacco, May 14, 2012*; 2012. Available at http://www.stevevape.com/wp-content/uploads/2012/05/Clearing-the-Smoke-on-E-Cigarettes.pdf.
3. Longhin D, Polchi V: **[The war of non-smoking against electronic cigarette] La Guerra del non fumo sulla sigaretta elettronica.** In *La Repubblica*; 2012. Available at http://www.repubblica.it/salute/prevenzione/2012/12/06/news/sigaretta_elettronica-48167614/.
4. Wembridge M, Thompson C: **Big Tobacco bets on e-cigarette future.** In *Financial Times*; 2012. Available at http://www.ft.com/cms/s/0/cb76997e-e0a5-11e1-b465-00144feab49a.html#axzz2JTedookH.
5. Etter JF, Bullen C: **Electronic cigarette: users profile, utilization, satisfaction and perceived efficacy.** *Addiction* 2011, **106**(11):2017–2028.
6. Goniewicz ML, Zielinska-Danch W: **Electronic cigarette use among teenagers and young adults in Poland.** *Pediatrics* 2012, **130**(4):e879–e885.
7. Pearson JL, Richardson A, Niaura RS, Vallone DM, Abrams DB: **e-Cigarette awareness, use, and harm perceptions in US adults.** *Am J Public Health* 2012, **102**(9):1758–1766.
8. McQueen A, Tower S, Sumner W: **Interviews with "vapers": implications for future research with electronic cigarettes.** *Nicotine Tob Res* 2011, **13**(9):860–867.
9. Freiberg M: **Options for state and local governments to regulate non-cigarette tobacco products.** *Ann Health Law* 2012, **21**(2):407–445. 405 p preceding i.
10. Etter JF, Bullen C, Flouris AD, Laugesen M, Eissenberg T: **Electronic nicotine delivery systems: a research agenda.** *Tob Control* 2011, **20**(3):243–248.
11. Caponnetto P, Campagna D, Papale G, Russo C, Polosa R: **The emerging phenomenon of electronic cigarettes.** *Expert Rev Respir Med* 2012, **6**(1):63–74.
12. WHO Study Group on Tobacco Product Regulation: **Report on the scientific basis of tobacco product regulation.** In *WHO Technical Report Series. Volume 955.* Edited by WHO; 2010. Available at http://www.who.int/tobacco/global_interaction/tobreg/publications/9789241209519.pdf.
13. *Regulation of E-Cigarettes and Other Tobacco Products.* http://www.fda.gov/NewsEvents/PublicHealthFocus/ucm252360.htm.
14. Draisci R, Vella S, Italian Institute of Health: *[Detalied report on the characteristics and functioning of electronic cigarettes] Relazione dettagliata sulle caratteristiche e sul funzionamento delle sigarette elettroniche.* Rome: Istituto Superiore di Sanità; 2012.
15. Gartner C: *ACTRN12612001210864 - Can using nicotine as a long-term substitute enhance smoking cessation over using it only as a cessation aid?.* Autralian New Zealand Clinical Trials Registry (ANZCTR). Available at http://apps.who.int/trialsearch/trial.aspx?trialid=ACTRN12612001210864.
16. Bullen C, McRobbie H, Thornley S, Glover M, Lin R, Laugesen M: **Effect of an electronic nicotine delivery device (e cigarette) on desire to smoke and withdrawal, user preferences and nicotine delivery: randomised cross-over trial.** *Tob Control* 2010, **19**(2):98–103.
17. Polosa R, Caponnetto P, Morjaria JB, Papale G, Campagna D, Russo C: **Effect of an electronic nicotine delivery device (e-Cigarette) on smoking reduction and cessation: a prospective 6-month pilot study.** *BMC Public Health* 2011, **11**:786.
18. Eissenberg T: *NCT00932295 - Evaluating the Acute Effects of Electronic Nicotine Delivery Devices Marketed to Smokers.* Clinicaltrials.gov.
19. Polosa R: *NCT01164072 - Efficacy and Safety of an Electronic Nicotine Delivery Device (E-Cigarette).* Clinicaltrials.gov.
20. Polosa R: *NCT01188239 - A Structured Protocol to Evaluate Efficacy and Safety of a Popular Electronic Nicotine Delivery Device (E-Cigarette) Efficacy and Safety of a Popular Electronic Nicotine Delivery Device (E-Cigarette).* Clinicaltrials.gov.
21. Goniewicz ML: *NCT01454362 - Effect of the Electronic Cigarette on Withdrawal Symptoms.* Clinicaltrials.gov.
22. West O: *NCT01714778 - Toxins and Delivery in e-Cigarette Users.* Clinicaltrials.gov.
23. Oncken S: *NCT01775787 - Effects of Electronic Cigarettes on Nicotine Concentrations.* Clinicaltrials.gov.
24. Vardavas CI, Anagnostopoulos N, Kougias M, Evangelopoulou V, Connolly GN, Behrakis PK: **Short-term pulmonary effects of using an electronic cigarette: impact on respiratory flow resistance, impedance, and exhaled nitric oxide.** *Chest* 2012, **141**(6):1400–1406.
25. Hajek P: *NCT01842828 - Spain-UK-Czech E-cigarette Study ((SUKCES)).* Clinicaltrials.gov.
26. Dawkins L, Turner J, Hasna S, Soar K: **The electronic-cigarette: effects on desire to smoke, withdrawal symptoms and cognition.** *Addict Behav* 2012, **37**(8):970–973.
27. Jarvis MJ, Tunstall-Pedoe H, Feyerabend C, Vesey C, Saloojee Y: **Comparison of tests used to distinguish smokers from nonsmokers.** *Am J Public Health* 1987, **77**(11):1435–1438.
28. Fritz M, Wallner R, Grohs U, Kemmler G, Saria A, Zernig G: **Comparable sensitivities of urine cotinine and breath carbon monoxide at follow-up time points of three months or more in a smoking cessation trial.** *Pharmacology* 2010, **85**(4):234–240.
29. *European Quality of Life Questionnaire.* http://www.euroqol.org/home.html.
30. Agency for Healthcare Research and Quality (AHRQ): *Prevention Quality Indicators, Technical Specifications, Version 4.3. August 2011 edition.* Agency for Healthcare Research and Quality (AHRQ); 2011. Available at http://www.qualityindicators.ahrq.gov/modules/pqi_resources.aspx.
31. Rabe-Hesketh S, Skrondal A (Eds): *Multilevel and longitudinal modelling using Stata.* 2nd edition. College Station: Stata Press; 2008.
32. Hosmer DW, Lemeshow S (Eds): *Applied Survival Analysis.* New York: John Wiley & Sons; 1999.

doi:10.1186/1471-2458-13-883
Cite this article as: Manzoli et al.: Multicentric cohort study on the long-term efficacy and safety of electronic cigarettes: study design and methodology. *BMC Public Health* 2013 **13**:883.

Submit your next manuscript to BioMed Central and take full advantage of:

- **Convenient online submission**
- **Thorough peer review**
- **No space constraints or color figure charges**
- **Immediate publication on acceptance**
- **Inclusion in PubMed, CAS, Scopus and Google Scholar**
- **Research which is freely available for redistribution**

Submit your manuscript at
www.biomedcentral.com/submit

BioMed Central
