# Supplementary material for: Electronic Cigarettes Efficacy and Safety at 12 Months: Cohort Study
Source: PLoS One. 2015 Jun 10;10(6):e0129443. doi: 10.1371/journal.pone.0129443 (PMC4464650; doi:10.1371/journal.pone.0129443)
Supplement: S2 File — (DOC) [file pone.0129443.s002.doc]

**Online supplemental Appendix**

**Outcome variables and data Analysis**

The differences in baseline values of the recorded variables by response status (non responders vs responders) and by baseline smoking status were evaluated using Kruskal-Wallis or one-way ANOVA with Sidak correction for continuous variables, and chi-squared test for categorical ones. For the latter variables, when more than two categories were to compare (such as for smoking status), separate comparisons were made for one group vs each other, and different p-values were computed. The difference in continuous variables (i.e. number of cigarettes smoked per day) within groups between baseline and end of follow-up were evaluated through Wilkoxon matched-pairs signed-ranks test.

The primary outcome was the percentage of subjects reporting sustained smoking abstinence from tobacco smoking at 12 months. Other outcomes were the proportion of quitters from all types of smoking, the number of tobacco cigarettes smoked, self-reported quality of life, and serious adverse events.

All variables were self-reported, with the exception of tobacco smoking cessation, that was tested using portable CO analyzers in a sub-sample of quitters and non-relapsing e-smokers. Adverse events data were also planned to be checked in the subjects from the Abruzzo Region through data linkage with hospital discharge abstracts and pharmaceutical administrative databases . However, Italian hospital and pharmaceutical administrative datasets are typically available on April of the following year, thus year 2014 data will not be available within the next months and could only be included in the report of the 24-month follow-up analysis. In the present analysis, the diseases occurred during the follow-up were self-reported (all participants were asked to report diseases "diagnosed by a physician"). The following diseases occurred during the 12-month follow-up were considered as "possibly related adverse events": Chronic obstructive pulmonary disease (COPD), Myocardial infarction and/or angina, Congestive heart failure, Transitory cerebrovascular ischemia or stroke, cancers of the: lung, esophagus, larynx, oral cavity, bladder, pancreas, kidney, stomach, cervix and myeloid leukemia. Additional possibly related serious adverse events were directly requested to the participants, but no subject reported any other event during the follow-up at this stage.

The self-rated health status was measured through a specific item of the Italian version of the validated EuroQol EQ-D5L questionnaire "How would you rate your current overall health status on a scale from 1 (worst imaginable) to 10 (best imaginable)?".

The outcomes that might have been influenced by a switch in smoking status during the follow-up (i.e. a smoker of tobacco cigarettes only switching to e-cigarettes only) were reported twice: by smoking status at baseline and by both smoking status at baseline and at 12 months, thus creating nine or twelve categories (according to whether the quitters of all smoking were to be included or not):

1. Baseline smokers of e-cigarettes only who quit all smoking (at 12 months);
2. Baseline smokers of e-cigarettes only who continued to smoke e-cigarettes only;
3. Baseline smokers of e-cigarettes only who continued to smoke e-cigarettes and also relapsed to tobacco smoking;
4. Baseline smokers of e-cigarettes only who ceased e-cigarette smoking and relapsed to tobacco smoking;
5. Baseline smokers of tobacco cigarettes only who quit all smoking;
6. Baseline smokers of tobacco cigarettes only who quit tobacco smoking and started e-cigarette smoking;
7. Baseline smokers of tobacco cigarettes only who continued to smoke tobacco cigarettes only;
8. Baseline smokers of tobacco cigarettes only who continued to smoke tobacco cigarettes and also started e-cigarette smoking;
9. Baseline smokers of both tobacco and e-cigarettes who quit all smoking;
10. Baseline smokers of both tobacco and e-cigarettes who quit tobacco smoking and continued to smoke e-cigarettes only;
11. Baseline smokers of both tobacco and e-cigarettes who quit e-cigarette smoking and continued to smoke tobacco cigarettes only;
12. Baseline smokers of both tobacco and e-cigarettes who continued to smoke both tobacco and e-cigarettes.

Multivariable random-effect linear and logistic regressions, with region as the cluster unit, were used to investigate potential predictors of continuous and categorical outcomes, respectively. We set four multivariable models for the following outcomes: (A) tobacco smoking abstinence at 12 months; (B) quit of all smoking; (C) difference in the daily number of tobacco cigarettes smoked per day between 12 months and baseline (smokers of e-cigarettes only at baseline were excluded from this model); (D) difference in the self-rated health from 12 months to baseline.

Given that with sparing use of dummy variables the number of successes of categorical outcomes approximated 10 for each recorded variable (thus fulfilling overfitting requirements), it was decided a priori to include all recorded variables into all final models regardless of significance, unless some multicollinearity or other incompatibility exist. Each covariate was tested in its original form or transformed if needed. In particular, the number of cigarettes smoked per day was also included as its square root with no changes, and it was thus kept in its original form. All final models were thus adjusted for the following baseline characteristics: age, gender, body mass index (BMI), marital status, educational level, occupation, alcohol use, hypertension, hypercholesterolemia, diabetes, self-rated health and years of tobacco smoking (former smoking for e-cigarette only smokers). We never included physical activity as a covariate in final multivariate models, due to the many missing data (n=69) and its virtually null effect on any dependent variables. The number of tobacco cigarettes smoked per day was also included in model C, while such variable was transformed to be included in models A, B and D: because no tobacco cigarettes were smoked at baseline by e-cigarette only users, we re-categorized the amount of smoking using tertiles. Those smoking at baseline less than 10 tobacco cigarettes per day (or <50 puffs per day if e-cigarette only smokers) were assigned to the lowest tertile of consume; the baseline smokers of 10-19 tobacco cigarettes (or 50-100 puffs if e-cigarette only users) per day were assigned to the intermediate tertile; baseline smokers of 20 or more cigarettes (or 100 or more puffs if e-cigarette only users) per day were classified into the highest tertile.

Potential interactions with the dependent variable and/or quadratic/cubic terms were investigated for all covariates. In logistic regression analyses, the outlier analysis was based upon the calculation of Pearson and standardized residuals, the change in Pearson chi-square and deviance chi-square, Dbeta influence statistic and leverage (hat diagonal matrix). The validity of the final linear regression models was assessed as follows. The assumption of constant error variance was checked graphically, plotting Pearson residuals vs. fitted values, and formally, using the Cook-Weisberg test for heteroskedasticity. High leverage observations were identified by computing Pearson, standardized and studentized residuals, Cook's D influence, Welsch distance and the hat diagonal matrix . We found 26 and 36 influential observations in the logistic models A and B, respectively, and 29 and 32 high-leverage observations in the linear models C and D, respectively. In all cases, we repeated the analyses excluding these observations, with no substantial changes, and we thus kept all observations into the models.

We had very few missing data for all variables (<5) except self-rated health, which was not answered at baseline by 56 participant, at 12 months by other 29 individuals. Models A, B and C were re-run without self-rated health at baseline (-56 subjects), with no appreciable variation, and such a covariate was thus retained. Model D was inevitably fit with 85 missing observations. Given that the 29 subjects lost in model D were relatively few (3.3% of the sample) and balanced across smoking groups, no missing data imputation technique was adopted. Finally, the distribution of the difference in self-rated health was relatively skewed (Shapiro-Wilk p<0.01) and model D was set also using its cubic form. However, again the estimates of p-values of smoking status covariates were similar and the dependent variable was maintained in its original form to facilitate results interpretation.

The results of the logistic analyses are presented as odds ratios (ORs) and the corresponding 95% confidence intervals (CIs) whereas the results of the linear regression analyses are presented as beta-coefficients and 95% CIs. A two-tailed p-value of 0.05 was considered significant for all analyses, which were performed using Stata 13.1 (Stata Corp., College Station, TX, USA, 2014).

**References**

1. Manzoli L, La Vecchia C, Flacco ME, Capasso L, Simonetti V, Boccia S, et al. Multicentric cohort study on the long-term efficacy and safety of electronic cigarettes: study design and methodology. BMC Public Health. 2013;13:883.

2. EuroQol Group. European Quality of Life Questionnaire. 2013 [February 1, 2013]; Available from: http://www.euroqol.org/home.html.

3. Hamilton LC, editor. Statistics with Stata: Version 12, Eighth Edition. Boston: Cengage; 2013.
