# Supplementary material for: Electronic Cigarettes Efficacy and Safety at 12 Months: Cohort Study
Source: PLoS One. 2015 Jun 10;10(6):e0129443. doi: 10.1371/journal.pone.0129443 (PMC4464650; doi:10.1371/journal.pone.0129443)
Supplement: S1 Table — (DOC) [file pone.0129443.s003.doc]

**Table S1**. Characteristics of the subjects completing the 12-month follow-up vs withdrawals or lost-to-follow-up subjects.

| **Variables** | **Completing the 12-month follow-up** | **Withdrawals or lost-to-follow-up** |  |
| --- | --- | --- | --- |
|  | *(N=959)* | *(n=396)* | *p** |
|  |  |  |  |
| Mean age in years (SD) | 44.5 (11.6) | 43.0 (11.6) | 0.035 |
| Male gender, % | 55.9 | 58.3 | 0.4 |
| Mean BMI (SD) | 24.6 (4.0) | 24.8 (3.9) | 0.4 |
| Married, % | 56.4 | 52.9 | 0.2 |
| Employed, % | 78.2 | 78.0 | 0.9 |
|  |  |  |  |
| *Educational level, %* |  |  |  |
| - Elementary / Middle | 22.4 | 25.7 | 0.2 |
| - High school | 46.3 | 47.3 | 0.7 |
| - Bachelor or higher | 31.3 | 27.0 | 0.11 |
|  |  |  |  |
| *Physical activity (69 missing)* |  |  |  |
| - At work, % | 18.9 | 23.2 | 0.083 |
| - Weekly hours at work, mean (SD) | 25.3 (17.5) | 27.1 (19.0) | 0.5 |
| - At home, % | 48.8 | 43.9 | 0.10 |
| - Weekly hours at home, mean (SD) | 5.3 (5.1) | 5.0 (3.8) | 0.5 |
|  |  |  |  |
| *Alcohol use* |  |  |  |
| Regular alcohol intake, % | 26.6 | 26.0 | 0.9 |
| Mean alcohol units daily (SD) | 2.1 (1.4) | 2.1 (1.6) | 0.9 |
|  |  |  |  |
| *Cardiovascular risk and health* |  |  |  |
| - Hypertension, % | 11.7 | 9.6 | 0.3 |
| - Diabetes, % | 3.8 | 3.0 | 0.5 |
| - Hypercholesterolemia, % | 9.0 | 6.3 | 0.10 |
| - Self-reported health, mean (SD) ^€^ | 7.8 (1.3) | 8.0 (1.3) | 0.10 |
| - Low self-reported health (<6) ^€^, % | 4.9 | 5.2 | 0.8 |
|  |  |  |  |
| *Smoking status, %* |  |  |  |
| - E-cigarettes only | 24.6 | 27.0 | 0.4 |
| - Tobacco cigarettes only | 51.2 | 51.0 | 0.9 |
| - Both tobacco and e-cigarettes | 24.2 | 22.0 | 0.4 |
|  |  |  |  |
| *Smoking pattern, mean (SD)* |  |  |  |
| - Years of tobacco smoking | 23.3 (12.6) | 22.3 (12.1) | 0.3 |
| - N. tobacco cigarettes daily | 14.4 (8.7) | 13.9 (8.1) | 0.4 |
| - Months of electronic smoking | 8.6 (4.8) | 8.7 (3.0) | 0.7 |
| - N. e-cigarette daily puffs | 130 (224) | 145 (313) | 0.5 |
| - E-cigarette nicotine dose in mg | 9.8 (5.5) | 10.2 (4.5) | 0.4 |
|  |  |  |  |
| - Former tobacco smoking, % | 100.0 | 99.5 | 0.9 |
| - Use of other tobacco products ^Ψ^, % | 0.6 | 1.3 | 0.3 |
| - Use of other nicotine products ^Ω^, % | 0.1 | 0.0 | 0.5 |
|  |  |  |  |
| *Reasons of e-cigarette smoking* ^φ^ |  |  |  |
| - Stop tobacco smoking, % | 60.0 | 56.7 | 0.4 |
| - Reduce tobacco smoking, % | 36.5 | 36.6 | 0.9 |
| - Indoor smoking, % | 14.1 | 10.3 | 0.2 |
|  |  |  |  |

^€^ EuroQol final item, ranging from 1 (feel very bad) to 10 (perfectly healthy). ^Ψ^ Cigars or tobacco chewing. ^Ω^ Nicotine patch or gums. ^φ^ More than one answer allowed.
