# Supplementary material for: Electronic Cigarettes Efficacy and Safety at 12 Months: Cohort Study
Source: PLoS One. 2015 Jun 10;10(6):e0129443. doi: 10.1371/journal.pone.0129443 (PMC4464650; doi:10.1371/journal.pone.0129443)
Supplement: S2 Table — (DOC) [file pone.0129443.s004.doc]

**Table S2**. Secondary outcomes.

|  | ***Baseline smoking status*** | | |
| --- | --- | --- | --- |
|  | **E-cigarettes**  **only** | **Tobacco**  **cigarettes only** | **Dual**  **smoking** |
|  | (n=236) | (n=491) | (n=232) |
|  |  |  |  |
| **1. Smoking pattern** |  |  |  |
|  |  |  |  |
| *Mean n. of tobacco cigarettes daily (SD)* |  |  |  |
| *At baseline* |  |  |  |
| - Started or continued tobacco cigarettes only | 0.0 (0.0) | 14.3 (7.9) | 15.4 (9.5) |
| - Started or continued dual smoking only | 0.0 (0.0) | 19.0 (6.3) | 14.0 (9.5) |
| *At 12 months* |  |  |  |
| - Started or continued tobacco cigarettes only | 16.0 (8.3) | 12.8 (7.6) | 16.1 (8.2) |
| - Started or continued dual smoking only | 8.0 (9.0) | 12.8 (7.2) | 9.3 (7.8) |
|  |  |  |  |
| % of subjects reducing tobacco cigarettes of 50% or more from baseline to 12 month |  |  |  |
| - Started or continued tobacco cigarettes only | -- | 16.4 | 9.2 |
| - Started or continued dual smoking only | -- | 44.4 | 40.0 |
|  |  |  |  |
| *% of subjects who smoked ≥5 tobacco cigarettes less between baseline and 12 months* |  |  |  |
| - Started or continued tobacco cigarettes only | -- | 25.2 | 26.1 |
| - Started or continued dual smoking only | -- | 77.8 | 44.0 |
|  |  |  |  |
| **2. Self-reported health** ^€^ |  |  |  |
|  |  |  |  |
| *Stratified by smoking status at baseline* |  |  |  |
| - Mean score (SD) | 8.2 (1.3) | 7.8 (1.5) | 7.9 (1.6) |
| - Mean difference vs baseline (SD) | +0.3 (1.5) | 0.0 (1.5) | +0.1 (1.7) |
| - Low (<6) score, % | 2.6 | 6.1 | 6.8 |
| - Improvement of ≥1 point, % | 40.9 | 30.9 | 38.0 |
|  |  |  |  |
| *Stratified by smoking status at 12 months* |  |  |  |
| Quit all smoking | *(n=36)* | *(n=56)* | *(n=22)* |
| - Mean score (SD) | 8.7 (1.1) | 8.0 (1.6) | 8.0 (2.0) |
| - Mean difference vs baseline (SD) | +0.3 (1.4) | +0.2 (2.0) | +0.7 (1.7) |
| - Low (<6) score, % | 0.0 | 5.4 | 9.1 |
| - Improvement of ≥1 point, % | 50.0 | 40.7 | 63.2 |
|  |  |  |  |
| Smoking e-cigarettes only | *(n=109)* | *(n=34)* | *(n=24)* |
| - Mean score (SD) | 8.4 (1.4) | 7.9 (1.9) | 8.6 (1.0) |
| - Mean difference vs baseline (SD) | +0.5 (1.3) | +0.9 (2.1) | +1.0 (1.4) |
| - Low (<6) score, % | 3.7 | 14.7 | 0.0 |
| - Improvement of ≥1 point, % | 47.5 | 57.6 | 54.5 |
|  |  |  |  |
| Smoking tobacco cigarettes only | *(n=61)* | *(n=376)* | *(n=121)* |
| - Mean score (SD) | 7.6 (1.2) | 7.8 (1.4) | 7.8 (1.5) |
| - Mean difference vs baseline (SD) | -0.4 (1.6) | -0.1 (1.4) | -0.1 (1.8) |
| - Low (<6) score, % | 3.3 | 5.0 | 5.8 |
| - Improvement of ≥1 point, % | 23.2 | 26.6 | 32.1 |
|  |  |  |  |
| Dual smoking | *(n=25)* | *(n=9)* | *(n=55)* |
| - Mean score (SD) | 8.2 (1.0) | 7.2 (1.4) | 7.6 (1.7) |
| - Mean difference vs baseline (SD) | +0.6 (1.3) | -0.1 (1.7) | +0.1 (1.5) |
| - Low (<6) score, % | 0.0 | 22.2 | 10.9 |
| - Improvement of ≥1 point, % | 43.5 | 33.3 | 35.2 |
|  |  |  |  |

SD = Standard deviation. ^€^ Last item of the EuroQol questionnaire, ranging from 1 (feel very bad) to 10 (perfectly healthy). This variable had 56 missing values at baseline, and another 29 at 12 months.
